# Low birth weight and associated factors in rural population of Rajasthan, India

**DOI:** 10.3389/fgwh.2025.1587991

**Published:** 2025-07-16

**Authors:** Ramesh Kumar Sangwan, Ramesh Kumar Huda, Mukti Khetan, Parul Gazta, Pankaj Kumar, Bontha V. Babu

**Affiliations:** ICMR-NIIRNCD, Jodhpur, India

**Keywords:** low-birth weight, social determinants, maternal health factors, nutrition and diet, behavioural health practices

## Abstract

**Background:**

Low Birth Weight (LBW) significantly affects childhood survival, with the socio-demographic characteristics (maternal age, child's gender, education, maternal diseases and others) contributing to it. The study aims to identify social determinants contributing to LBW, which can further be useful in developing local interventions to rectify the problem in an Indian rural context.

**Methodology:**

The cross-sectional study was conducted in the Jalore district of Rajasthan, India. A total of 92 delivery cases, including LBW (*n* = 46) and cases with normal birth weight (*n* = 46), became part of the research. A pre-tested questionnaire collected information from study participant groups enumerating deliveries from selected Primary Health Centres (PHCs) related to LBW and non-LBW deliveries in a 1:1 ratio.

**Results:**

The study recorded a total of 1,251 deliveries, of which 63 resulted in the LBW (<2,500 grams), nine were premature, 12 were twin births, and 361 were normal weight deliveries (≥2,500 grams). LBW was prevalent in underprivileged communities within nuclear families, having an average birth weight of 2.12 kilograms. Reduced meal frequency (1–2 times a day) for women is also linked to higher LBW risk.

**Conclusion:**

Many factors, like complications during pregnancy, awareness of pregnancy planning, and nutritional intake, are associated with the likelihood of LBW occurrences. Many maternal risk factors for LBW are modifiable through early detection by imparting education and awareness to pregnant women in their first trimester. The findings emphasize the significance of targeted interventions and awareness programs to address specific risk factors and improve birth outcomes in rural Indian communities.

## Introduction

Birth weight serves as both a crucial measure of maternal well-being and a strong predictor of neonatal and childhood health outcomes. The newborn's birth weight is ideally documented within the first hours of delivery and captured before significant postnatal weight loss takes place. A newborn with low birth weight (LBW) is characterized as a live baby weighing less than 2,500 grams at birth. According to WHO estimates, approximately 25 million babies with LBW are born annually, and 5 million of them experience global mortality (1). According to the National Family Health Survey (NFHS-5) (2), the Infant Mortality Rate (IMR) in India was reported to be 26.6%. The LBW babies are at a higher risk of mortality within their first month, and those who survive confront lifelong consequences (3, 4). The public health data reveals that 98.8% of live births were recorded in India, with Rajasthan having a percentage of 98.2%. The LBW rate was recorded as 14.5% in Rajasthan (5). The NFHS-5 records a prevalence of 18.2% LBW in India, while in Rajasthan, it was 17.7% (2).

A child's birth weight is a critical determinant of susceptibility to childhood illnesses and overall survival. It is a widely accepted measure for assessing health status and is a key focal point in health policy discussions (6). Low birth weight (<2,500 grams) babies face a twenty-fold higher risk of neonatal death compared to normal weight babies (≥2,500 grams) (7). LBW is strongly linked to fetal and neonatal mortality, morbidity, impaired growth, cognitive development, as well as the onset of chronic diseases in later life. The impact of LBW on cardiovascular risk and metabolic disorders among children, adolescents, and young adults is noteworthy. During early childhood, LBW babies may exhibit heightened blood pressure, impaired vascular growth, increased peripheral vascular resistance, and cardiomyocyte remodeling (8).

Numerous factors interconnected with the infant, the mother, and the physical environment influence the duration of gestation and fetal growth, further impacting birth weight (9). These include tobacco exposure, inadequate antenatal care, maternal hypertension, low socioeconomic status, maternal anaemia, prenatal care visits, and maternal education. The risk of delivering an LBW baby is 4.1 times higher in women exposed to any tobacco product compared to those without exposure (10). Socio-cultural factors inevitably shape the healthcare vulnerability of rural populations (11). Limited awareness about healthcare measures and a lack of education and resources often contribute to inadequate health-seeking behaviour and poor health outcomes. Despite several government efforts to improve maternal health over the last two decades, disparities persist between various social groups. Identifying factors associated with the risk of LBW allows for targeted interventions, such as counselling mothers when feasible. Addressing these factors can reduce perinatal mortality and morbidity. It is imperative to enhance the quality and utilization of antenatal care, offer nutritional education for improved pregnancy weight gain, advocate for proper spacing, discourage tobacco use, and effectively manage risk factors like anaemia and hypertension (12). This study aims to identify maternal and socio-demographic factors associated with the risk of LBW deliveries in rural Rajasthan, using a matched case-control design.

## Methodology

This cross-sectional study was conducted in Ahore Block of Jalore district, Rajasthan, from February 2021 to August 2022. As per Census of India 2011, the district has a population of 1,828,730, of which Ahore block contributes 239,642 population with a low literacy rate and economic backwardness. There are 10 primary healthcare centres (PHCs) under the district's primary healthcare system. Out of these, two PHCs-Bhadrajun (with five sub-health centres) and Gudhabalotan (with nine sub-health centres) became part of the current research. The required primary information on childbirths and the status of pregnant women and would-be mothers was gathered from these sub-health centres (SHCs).

### Sample size

As preterm birth is one of the major factors associated with LBW, an estimation of its prevalence was taken as a basis for the sample size to be covered in the hospital survey. Assuming 10% as the preterm babies, at a 5% level of significance and 10% relative precision, about 900 deliveries were enumerated to estimate the point prevalence of preterm babies. 1,251 deliveries were recorded during the study, including 63 deliveries resulting in LBW. Of 63 LBW deliveries, three denied consent to participate in the study, and 14 were found unavailable, resulting in a total of 46 cases that became part of the study sample. To compare and contrast, a similar number of 46 deliveries with non-LBW babies were selected, considering a similar case match based on the age and locality of the participant.

### Inclusion and exclusion criteria

The study includes mothers of live-born singleton term babies, with cases defined as birth weights under 2,500 grams (<2,500 grams) as well as 2,500 grams or more (≥2,500 grams). Exclusions were incomplete records, congenital anomalies, twin births, uncontactable cases after three home visits, and home deliveries.

### Data collection

A pre-tested questionnaire was utilized to interview and collect the required information from the eligible mothers (study participants) through face-to-face conversation. The content was developed in the local language to understand the respondents better. The collected data was then translated into English with the help of a language expert to communicate findings in a research paper form. The variables under study include the basic socio-demographic profiles and the personal and obstetric history of the participants.

### Data analysis

Data was analyzed using SPSS V.28. In addition to descriptive statistics, the Least Absolute Shrinkage and Selection Operator (LASSO) logistic regression was used to identify the factors associated with the birth of LBW babies. In this study, the authors have utilized R version 4.3.1 to perform logistic LASSO regression to identify the key predictors of LBW. They adjusted the lambda sequence to avoid very small values and fitted the logistic LASSO regression model with the *glmnet package*. The optimal lambda value was identified through cross-validation using the *cv.glmnet function*, and the best lambda was selected based on the minimum cross-validation error. The authors performed bootstrapping with 1,000 iterations to calculate the coefficients’ standard errors.

## Results

Table 1 presents the PHC-wise details of deliveries reported. 1,251 deliveries were recorded from two PHCs, including 63 deliveries resulting in LBW, nine premature, 12 twin births, and 361 normal weight deliveries (≥2,500 grams). The average weight of LBW babies was 2.12 kg, while it was 3 kg for normal healthy babies. Out of 1,251 recorded deliveries, 36.69% (*n* = 459) took place in government institutions, 58.43% (*n* = 731) in private health facilities, and 4.88% (*n* = 61) occurred at home.

**Table 1:** Primary health centre-wise details of deliveries reported.

Particulars	PHC Bhadrajun (n)	PHC Gudha Balotan (n)	Total (n)	Total (%)
Based on the delivery location				
Government hospital	171	288	459	36.69
Private hospital	297	434	731	58.43
Home	34	27	61	4.88
Based on Birth Weight				
Low birth cases	16	47	63	5.04
The birth weight recorded 2,500 grams	177	184	361	28.86
Birth weight recorded >2,500 grams	303	503	806	64.43
Preterm cases	2	7	9	0.72
Twin Birth Cases	4	8	12	0.96
Total	502	749	1,251	100.00

PHC, primary health centre.

Table 2 shows the Socio-demographic characteristics of mothers. The average age of the respondents was 26.5 years. The average age of women at marriage was 17.5 years in the case of LBW deliveries and 19 years recorded for normal deliveries. It was found that the average age of the women at the first childbirth was 20 years, compared to 21 years for women with normal weight deliveries. A variation was seen in family annual income, where households with LBW cases recorded an income of Indian rupees (INR) 2.1 lakh, whereas it was INR 2.5 lakh for the other group. LBW incidences were recorded as higher among labourers. The study results show that LBW incidents were higher in nuclear families.

**Table 2:** Socio-demographic characteristics of mothers.

Particulars		Women with the birth of LBW babies		Women with the birth of normal baby	
		*n* = 46	%	*n* = 46	%
The average age of the woman		26.5 years		26.5 years	
The average age at marriage		17.5 years		19 years	
Average age at first childbirth		20 years		21 years	
Average total family members		7 members		7 members	
Average annual family income (in INR)		2.1 Lakh		2.5 lakh	
Occupation of mothers	Job	0	0.00	2	4.35
	Farmer	2	4.35	2	4.35
	Homemaker	31	67.39	38	82.61
	Labour	13	28.26	4	8.70
Education of spouse (years of schooling)	No schooling	11	23.91	2	4.35
	1–5	5	10.87	5	10.87
	6–10	20	43.48	29	63.04
	12	10	21.74	10	21.74
Social category	General	7	15.22	7	15.22
	Backward community	13	28.26	23	50.00
	Scheduled caste	13	28.26	14	30.43
	Scheduled tribe	13	28.26	2	4.35
Type of family	Extended	34	73.91	41	89.13
	Nuclear	12	26.09	5	10.87
Weight of the baby at the time of birth	Average weight (in kilograms)	2.12		3.00	

INR, Indian Rupees (1 INR = 0.012 US $).

Table 3 compares case (LBW babies) and control (non-LBW babies) groups, evaluating various factors influencing low birth weight. However, notable associations were observed in several other factors. Being diagnosed with any disease showed a significant link with LBW occurrences. Additionally, variables such as awareness of pregnancy planning, history of miscarriage, complications in pregnancy, daily meals taken, fruit consumption, extra protein intake, and pressure during pregnancy exhibited statistically significant associations with LBW. The findings suggest that, although some factors may not exhibit a strong association with LBW, others, such as being diagnosed with diseases during pregnancy, awareness of pregnancy planning, nutritional intake, and specific pregnancy-related complications, may have the potential to influence the likelihood of LBW occurrences.

**Table 3:** Details of the association of mothers’ characteristics with LBW.

Variable	Category	Case	Control	P-Value
Education	Non-formal (up to 5th)	32	21	0.20
	Formal education (6–12th)	14	25	
Place of delivery	Government hospital	25	19	0.21
	Private hospital	21	27	
Gender of the baby	Male	24	25	0.83
	Female	22	21	
Baby diagnosed with any disease	Yes	8	0	0.003*
	No	38	46	
Awareness of the gap between children	Yes	31	36	0.24
	No	15	10	
Planned Pregnancy	Yes	25	40	0.001
	No	21	6	
Aware of Antenatal care	Yes	42	45	0.36*
	No	4	1	
Taken antenatal care	Yes	44	45	1.00*
	No	2	1	
Miscarriage or abortion	Yes	31	13	0.00
	No	15	33	
Complications in current pregnancy	Yes	5	0	0.05*
	no	41	46	
Tobacco use by mothers	Yes	12	4	0.28
	No	34	42	
Alcohol consumption by mother	Yes	1	0	1.00
	No	45	46	
Daily meals consumption	3–4 times	30	9	0.00
	1–2 times	16	37	
Fruit intake during pregnancy	Yes	22	38	0.00
	No	24	8	
Non-vegetarian food consumption by mother	Yes	6	6	1.00
	No	40	40	
Supplementary protein intake by mother	Yes	37	46	0.003*
	No	9	0	
Rest during pregnancy by mother	Yes	39	45	0.05
	No	7	1	
Tea or coffee consumption by mother	Yes	44	40	0.26*
	No	2	6	
Pressure for pregnancy	Yes	31	9	0.00
	no	15	37	
Place of stay during pregnancy	Maternal side	10	7	0.42
	In-laws side	36	39	
Place or stay after delivery	Maternal side	11	11	1.00
	In-laws side	35	35	

ANC, antenatal care.

* Fisher exact test *p* < 0.05.

### Factors associated with the birth of low-birth weight babies

Table 4 presents the LASSO logistic regression analysis results, highlighting the factors influencing LBW. It is utilized to handle multicollinearity and perform variable selection, especially given the number of predictors. LASSO helps identify the most relevant variables by shrinking some coefficients to zero, improving model interpretability. The significant coefficients identified in the model highlight several vital predictors of LBW. Babies diagnosed with chronic diseases have higher odds of being born with LBW, as indicated by a coefficient of 1.2512 (SE: 0.7508, CI: 0.0000, 2.4239). Pregnancies complicated by medical issues are strongly associated with higher odds of LBW, with a coefficient of 2.0861 (SE: 0.7552, CI: 0.0000, 3.2753). Women experiencing pressure to conceive are more likely to have LBW babies, as shown by a coefficient of 0.9670 (SE: 0.5761, CI: 0.0000, 2.0059). Reduced meal frequency (1–2 times a day) correlates with higher LBW risk, with a coefficient of 1.0436 (SE: 0.5882, CI: 0.0000, 2.2391). A history of miscarriage or abortion is associated with increased odds of LBW (coefficient: 0.8377, SE: 0.4931, CI: 0.0000, 1.8658). Marrying at an age younger than 18 years is linked to higher LBW risk, indicated by a coefficient of 0.5546 (SE: 0.5101, CI: 0.0000, 1.7349). Variables, such as maternal education, place of delivery, awareness about the gap between the pregnancies, ANC awareness, tobacco use, fruit intake, rest during pregnancy, tea or coffee consumption, occupation of women, type of family and social support, were not selected by the LASSO model and suggested that these factors do not have a significant association with LBW in this context. Other variables showed no clear association with LBW risk.

**Table 4:** Details of lasso regression coefficients for the association of mothers’ characteristics with LBW .

Variable	Category	Coefficient ± SE
Intercept		−1.8562 ± 0.5561
Education	Non-formal (up to 5th)	Reference
	Formal education (6–12th)	NS
Place of delivery	Private Hospital	Reference
	Government Hospital	NS
Diagnosed with any disease	No	Reference
	Yes	1.2512 ± 0.7508
Awareness of the gap between children	Yes	Reference
	No	NS
Planned Pregnancy	Yes	Reference
	No	0.0089 ± 0.3958
Aware of Antenatal care	Yes	Reference
	No	NS
Miscarriage or abortion	No	Reference
	Yes	0.8377 ± 0.4931
Complications in current pregnancy	No	Reference
	Yes	2.0861 ± 0.7552
Tobacco use by mothers	No	Reference
	Yes	NS
Fruit intake during pregnancy	Yes	Reference
	No	0.01239 ± 0.3714
Supplementary protein intake by the mother	Yes	Reference
	No	0.5270 ± 0.3849
Rest during pregnancy by the mother	Yes	Reference
	No	NS
Tea or coffee consumption by the mother	1. No	Reference
	2. Yes	NS
Pressure for pregnancy	No	Reference
	Yes	0.9670 ± 0.5761
Daily meals consumption	3–4 times	Reference
	1–2 times	1.0436 ± 0.5882
Age at marriage	≥=18	Reference
	<18	0.5546 ± 0.5101
Occupation of woman	Homemaker	Reference
	Labour	NS
	Others	NS
Social category	General	Reference
	Backward community	NS
	Scheduled caste	1.0388 ± 0.7242
	Scheduled tribe	NS
Type of family	Extended	Reference
	Nuclear	NS
Social Support	Yes	Reference
	No	NS
Number of living room	>2	Reference
	2	−0.0831 ± 0.3052
	1	NS
Spouse Education (years of schooling)	12	Reference
	6–10	NS
	1–5	NS
	No schooling	0.0222 ± 0.3182
Number of Siblings	0–1	Reference
	2	−0.3107 ± 0.4117
	>2	NS
Family income (in INR)	>3,00,000	Reference
	1,00,000–3,00,000	NS
	>1,00,000	0.5177 ± 0.4783

NS, non-significant; SE, standard error; INR; Indian rupees (1 INR = 0.012 US $).

## Discussion

The current research provides insights into LBW and its associated factors in rural Rajasthan. The study provides a comparison between LBW and non-LBW cases as per a pre-defined inclusion and exclusion criteria. LBW was more common among labourers recognized as underprivileged communities, especially in nuclear families. Similar results were documented by previous research (13) showing that mothers from lower socioeconomic strata and disadvantaged populations are known to have higher occurrences of LBW. Factors such as disease diagnosis during pregnancy, awareness of pregnancy planning, nutritional intake, and specific complications influenced the likelihood of LBW occurrences. Results revealed a strong association between a history of miscarriage and such outcomes. LBW is strongly associated with preterm birth. Mothers with a familial history of preterm birth face a higher risk. Maternal education strongly correlates with the frequency of preterm birth and LBW instances. Low socio-economic conditions increase the risk of LBW deliveries compared to better conditions. Infections during pregnancy are a major cause of LBW cases. Body Mass Index (BMI) plays a crucial role in LBW birth, and an interpregnancy interval of less than one year significantly impacts LBW birth rates. Mothers using multivitamins and folic acid during pregnancy have lower chances of LBW birth, while malnourished women are at a higher risk (14). The postponement of prenatal care initiation can be attributed to factors such as a lack of access to health services, maternal age, parity, or socioeconomic status. Economic hardship hinders individuals from accessing medical services, travelling for referrals, and acquiring essential food and sanitary products. These challenges, in turn, indirectly impact a baby's birth weight (15).

Mother's age, educational attainment and socioeconomic status greatly impact LBW in India (13). The adolescent mothers are more likely to have LBW babies (16). Confirming the same, the current research reiterates that marrying at an age younger than 18 years is linked to higher LBW risk. Among the examined socioeconomic factors, including maternal illiteracy, manual labour, nuclear family structure, poor socioeconomic status, consanguinity history, and tobacco consumption, a notable proportion of LBW cases was observed in mothers aged below 20 and above 30 years. Risk factors associated with LBW encompass elements such as insufficient iron intake (less than 180 tablets), inadequate weight gain (less than 6.53 kg) during the second and third trimesters, presence of comorbidities during pregnancy, attendance at antenatal care, and experiencing preterm birth. The literature emphasizes that various socio-demographic factors continue to play a crucial role in causing LBW among newborns (9). This underscores LBW as a complex, multi-faceted phenomenon. Mother's education, parity, pregnancy planning, twin birth and maternal smoking during pregnancy were significant determinants of LBW in developing nations (Islam and Khan, 2016; Islam et al., 2020) Furthermore, maternal health conditions such as hypertension, diabetes, and anaemia were identified as significant risk factors for delivering LBW babies, as highlighted in a study conducted in Kerala (17). Among the reported birth weights, 22 per cent of children had LBW, indicating a weight below 2.5 kg (17). The research also shows significant evidence highlighting that pregnancies complicated by medical issues and women experiencing pressure to conceive are more likely to have LBW babies (18). It found that the gender of the baby, type of family, socioeconomic status, maternal educational level, maternal occupation, anaemia, and iron-folic acid intake significantly influenced LBW in rural contexts in India. LBW prevalent in Northern India has been linked to infant deaths (19), finding that previous LBW is the strongest indicator for subsequent delivery of an LBW baby and may further exacerbate the risk of other adverse perinatal outcomes. They also highlight the importance of utilizing multiple co-occurring risk factors and the impact of compounding risks in determining preterm birth and LBW.

The present study suggests using educational interventions focusing on promoting national programmes, i.e., Poshan Abhiyaan, Pradhan Mantri Surakshit Matritva Abhiyan and Integrated Child Development Services (ICDS), for emphasis on nutrition and comprehensive and quality antenatal care for pregnant women and mothers. The ICDS scheme supports pregnant women by providing supplementary nutrition, antenatal care and health education through Anganwadi centres. It ensures better maternal health by addressing malnutrition, immunisation, and promoting institutional deliveries. These efforts help improve birth outcomes, especially in rural and underserved areas (19). Prioritizing policies to address LBW risk factors is crucial for significantly reducing infant mortality. Utilizing the media to raise awareness about LBW complications and implementing WHO-recommended public-private partnerships in the health sector can enhance survival outcomes for newborns with LBW (20). The factors can be effectively prevented through simple family actions, and mothers can easily adopt them. Maternal health programs should focus on encouraging and monitoring complete iron tablet intake during pregnancy. Families can support mothers by ensuring adequate rest, nutrition, and healthy behaviour to mitigate the identified risk factors (21). In some communities, pregnant women are restricted from eating sufficient food, not only due to poverty but also because of cultural practices or beliefs (22). There is a need for nationally representative data on the prevalence of LBW generated from community-based surveys (23–26). Future research should examine indicators like BMI, anaemia, smoking, alcohol use, and history of LBW during pregnancy. Tailored home-based neonatal care setups and revised awareness campaigns are essential for LBW babies (16). Establishing a portal for LBW at birth, recording weight, and providing supervision up to age five can help prevent under-5 mortality (16). Interventions targeting improvements in antenatal care access, maternal health, and nutritional status may help deal with the problem of LBW babies (13).

### Strengths and limitations

While discussing the study's implications, it is essential to highlight its strengths and limitations. The study involved a large sample size to strengthen the reliability of the findings. It provided a detailed analysis of various maternal, socio-economic and health-related factors influencing LBW occurrence. However, the study design is cross-sectional, which limits its ability to establish causal relationships. The design discloses the correlation but doesn’t establish cause and effect among the variables under study. As a result, the associations identified in this study should be interpreted as correlations rather than causal links. Another limitation may persist due to a possible recall bias, since many responses are self-reported by the study participants. Furthermore, the study does not account for home deliveries or the associated cases of LBW, potentially missing important data relevant to the findings. Incorporating a limited number of participant cases (*n* = 92) could impact the generalizability of the study findings.

## Conclusions

LBW was more prevalent in underprivileged communities and particularly in nuclear families. The average weight of LBW babies was 2.12 kg, compared to 3 kg in the healthy control group. Factors like disease diagnosis during pregnancy, awareness of pregnancy planning, nutritional intake, and specific complications were found to impact the likelihood of LBW occurrences. The significant coefficients identified in the model highlight several vital predictors of LBW. Pregnancies complicated by medical issues and women experiencing pressure to conceive are more likely to have LBW babies. Babies diagnosed with chronic diseases and reduced meal frequency for women (1–2 times a day) and marrying at an age younger than 18 years correlate with higher LBW risk. A history of miscarriage or abortion is strongly associated with increased odds of LBW. By minimizing the burden of LBW, India may progress towards achieving the Sustainable Development Goals target of reducing child mortality and malnutrition by 2030.

## Data Availability

The original contributions presented in the study are included in the article; further inquiries can be directed to the corresponding author.
